# The Gametocytes of *Leucocytozoon sabrazesi* Infect Chicken Thrombocytes, Not Other Blood Cells

**DOI:** 10.1371/journal.pone.0133478

**Published:** 2015-07-28

**Authors:** Wenting Zhao, Jianwen Liu, Ruixue Xu, Cui Zhang, Qin Pang, Xin Chen, Shengfa Liu, Lingxian Hong, Jing Yuan, Xiaotong Li, Yixin Chen, Jian Li, Xin-zhuan Su

**Affiliations:** 1 State Key Laboratory of Cellular Stress Biology, Innovation Center for Cell Signaling Network, School of Life Sciences, Xiamen University, Xiamen, Fujian, 361005, P. R. China; 2 National Institute of Diagnostics and Vaccine Development in Infectious Diseases, School of Public Health, Xiamen University, Xiamen, Fujian, 361005, P. R. China; 3 Laboratory of Malaria and Vector Research, National Institute of Allergy and Infectious Diseases, National Institutes of Health (NIH), Bethesda, Maryland, 20892, United States of America; Institute of Tropical Medicine, JAPAN

## Abstract

*Leucocytozoon* parasites infect a large number of avian hosts, including domestic chicken, and cause significant economical loss to the poultry industry. Although the transmission stages of the parasites were observed in avian blood cells more than a century ago, the specific host cell type(s) that the gametocytes infect remain uncertain. Because all the avian blood cells, including red blood cells (RBCs), are nucleated, and the developing parasites dramatically change the morphology of the infected host cells, it has been difficult to identify *Leucocytozoon* infected host cell(s). Here we use cell-type specific antibodies to investigate the identities of the host cells infected by *Leucocytozoon sabrazesi* gametocytes. Anti-RBC antibodies stained RBCs membrane strongly, but not the parasite-infected cells, ruling out the possibility of RBCs being the infected host cells. Antibodies recognizing various leukocytes including heterophils, monocytes, lymphocytes, and macrophages did not stain the infected cells either. Antisera raised against a peptide of the parasite cytochrome B (CYTB) stained parasite-infected cells and some leukocytes, particularly cells with a single round nucleus as well as clear/pale cytoplasm suggestive of thrombocytes. Finally, a monoclonal antibody known to specifically bind chicken thrombocytes also stained the infected cells, confirming that *L*. *sabrazesi* gametocytes develop within chicken thrombocytes. The identification of *L*. *sabrazesi* infected host cell solves a long unresolved puzzle and provides important information for studying parasite invasion of host cells and for developing reagents to interrupt parasite transmission.

## Introduction

*Leucocytozoon* is a genus of parasitic protozoa that belongs to the phylum of Apicomplexa. It contains over 100 species infecting more than 100 species of birds, including domestic chickens [1–7]. The life cycles of these parasites are similar to those of *Plasmodium* and *Haemoproteus* species and involve two hosts, with merogony in fixed tissues and sexual differentiation (gametocytes) in blood cells of avian hosts and sporogony in the midguts of simuliid flies or culicoides midges [1, 8]. Sporozoites in the salivary glands of an infected *Simulium* fly (blackfly) are injected into a host when the insect bites the bird. The injected sporozoites travel to the liver and develop into trophozoites and schizonts in hepatocytes. Mature merozoites are released and are believed to infect many types of blood cells including red blood cells (RBCs), leukocytes, macrophages or even endothelial cells [1]; however, it has been difficult to determine whether the parasites infect RBCs or white blood cells (WBCs). Parasites that infect macrophages or endothelial cells can develop into megaloschizonts, generating more merozoites. In response to unknown cues, some of the parasites develop into male and female gametocytes after invading some specific blood cells, and for many species, the gametocytes also transform the host cells into enlarged fusiform (tapering at both ends or spindle-shaped) cells. When a blackfly bites and takes blood from an infected bird containing mature gametocytes, male and female gametes fuse to form zygotes in the midgut within a few minutes [8]. The zygotes then developed into ookinetes that penetrate the midgut wall of the fly and mature into oocysts containing sporozoites that migrate to the salivary glands of the fly, completing the life cycle.

Compared with those of vertebrates, avian blood cells have some unique characteristics [9]: In addition to nucleated RBCs, avian blood cells include heterophils that are equivalent of mammalian neutrophils and play an important role in host immune defense [10]. The heterophils are large cells with segmented nuclei that are partly obscured by the large refractile granules in their cytoplasm. Another unique feature of avian blood is the nucleated thrombocytes that develop in the bone marrow and are functionally equivalent to mammalian platelets [11, 12]. A mature thrombocyte contains round or oval nucleus with densely clumped chromatin and a small rim of cytoplasm, whereas immature thrombocytes may have moderately abundant cytoplasm with at least one of the following features: colorlessness, vacuoles, and pink to magenta-colored granules after staining with Giemsa or Wright stain [9, 13]. Besides functioning in blood clotting [14], thrombocytes have been shown to have phagocytic activities [15, 16] and to play a role in inflammation [17, 18]. Avian lymphocytes in many ways are similar to thrombocytes, but they generally have larger nuclei with limited cytoplasm [9, 13]. Eosinophils, basophils, monocytes, and macrophages have lobed nuclei and granulated cytoplasm [9]. In theory, the parasites can infect any of the blood cells.

The diagnosis of *Leucocytozoon* infection is largely based on the observation of gametocytes in the blood smear of an infected bird or, more recently, PCR-based DNA detection [19, 20]. Traditionally, parasites were observed inside host blood cells, either RBCs or WBCs, after staining with specific dyes such as Giemsa or Wright stains. Because the parasites dramatically alter the morphology of the infected host cells, and the RBCs are nucleated, it has been difficult to determine the type(s) of blood cells in which *Leucocytozoon* gametocytes develop. It appears that the parasites generally infect blood cells with single nucleus, not the cells with multi-lobed nuclei. The question of whether *L*. *sabrazesi* (or other *Leucocytozoon* parasites) infects RBCs and/or any specific WBCs remains to be answered. To better understand the parasite biology, host-parasite interaction and the molecular basis of *Leucocytozoonosis*, we investigated the host blood cell type(s) that harbors *L*. *sabrazesi* gametocytes using antibodies specific for various chicken blood cells from infected chickens. Our results show that the gametocytes of *L*. *sabrazesi* develop specifically within thrombocytes and provide critical information for further studies of host-parasite interaction of this important avian pathogen.

## Materials and Methods

### Animals and blood collection

Infected blood samples were obtained from chickens in a free-range farm (Tzu Chi Chicken Farm) in Haicang district, Xiamen city, Fujian province of China (coordinates: 24°29′06.4″N 118°02′29.7″E). No endangered or protected species were used in this study. Blood was collected (200–300 μl) from the wing veins and was immediately mixed with an anticoagulant solution (8 g/L citric acid and 22 g/L trisodium citrate, pH 7.2) in 1.5 ml centrifuge tube. The blood samples were washed 2X with PBS at 3000 rpm for 3 min. Blood collection was performed after purchasing the animals from the farm owner; no specific permissions were required for these activities. For initial parasite identification, thin blood smears were made, air-dried, fixed with 100% methanol, and stained with Giemsa stain before microscopic examination. The experiments and sampling procedures were conducted under the protocol (#XMULAC20130065) reviewed and approved by the Animal and Care Ethics Committee at Xiamen University.

### Anti-blood cell antibodies used in this study

The following antibodies were used in this study: Mouse anti-chicken CD4 (CT-4) that stains thymocytes (70%), spleen cells (10%), peripheral blood lymphocytes (45%), and bursal cells (<1%); mouse anti-chicken Bu-1b (5-11G2) that stains bursal cells, thymocytes, monocytes and macrophages; and mouse anti-chicken monocyte/macrophage (anti-monocyte, KUL01) that stains monocytes and macrophages as well as interdigitating cells and activated microglia cells. These antibodies were purchased from Southern Biotech (Birmingham, AL). Rabbit anti-chicken RBC antibody (103–4139) was obtained from Rockland Immunochemicals, Inc. (Gilbertsville, PA). Mouse anti-human CD51/CD61 (clone 23C6) also specific for chicken thrombocytes was obtained from AbD Serotec (Raleigh, NC). Alexa Fluor 555 goat anti-mouse IgG and Alexa Fluor 555 goat anti-rabbit IgG were purchased from Life Technologies (Shanghai, China).

### Production of anti-*Leucocytozoon* CYTB antibodies

Mouse anti-*Leucocytozoon* cytochrome B (CYTB) was generated in our laboratory after immunization of BALB/c mice with a keyhole limpet hemocyanin (KLH) conjugated peptide (CYTB: SKRLHYDYSSQAN) that was derived from *L*. *sabrazesi* CYTB sequence we obtained previously [21]. Three mice were injected with 30 μg antigen in PBS after mixing with Freund’s complete adjuvant, followed with three boosters of the same amount of antigen in Freund’s incomplete adjuvant. Immunized sera were collected two weeks after final immunization.

### Immunofluorescence assay (IFA)

Blood samples were diluted in PBS (1:1000) before being applied to poly-L-Lysine- coated coverslips. After application of poly-L-Lysine (0.125%), coverslips were allowed to dry at RT for 10 min. The coverslips were washed twice with distilled water and air-dried again before addition of 400 μl of diluted blood sample. The blood sample was allowed to sit for 30 min at RT and then washed 2X with PBS plus gentle shaking for 5 min each. The cells were fixed with 4% formaldehyde at RT for 30 min, washed once with PBS, and incubated with 0.1% Triton X-100 at RT for 10 min. After Triton X-100 treatment, the cells were washed 2X in PBS, blocked with blocking buffer (3% BSA in PBS) for 2 h at RT or overnight at 4°C. For mouse anti-chicken CD4, mouse anti-chicken Bu-1b, and mouse anti-chicken monocyte/macrophage conjugated with fluorescein (FITC), the antibodies were diluted at 1:1,000 in blocking buffer, incubated for 2 h at RT with shaking, and washed 3X with blocking buffer for 10 min each with shaking. In the first washing, Hoechst dye (1:10,000) was included in the washing buffer. The coverslips were mounted with 90% glycerinum and sealed with Nail polish. The rabbit anti-chicken RBC antibody was diluted at 1:2,000 with blocking buffer and incubated at 4°C overnight. After washing 3X, Alexa Fluor 555 labeled goat anti-rabbit antibody diluted at 1:3,000 in blocking buffer was added and incubated at RT for 1 h. The wash and slide mounting procedures were the same as above. The procedures for the mouse anti-*Leucocytozoon* CYTB and anti-CD51/CD61 were the same as anti-chicken RBC, except that the primary antibody was diluted at 1:1000 (anti-CYTB) or 1:20 (anti-CD51/61) in blocking buffer, and Alexa Fluor 555 goat anti-mouse IgG was used as the secondary antibody. Alternatively, blood cells were washed in PBS 2X, and the smears were fixed in 100% cold methanol (-80°C) for 20 min. The slides were air-dried for 20 min before being treated with blocking buffer and with various antibodies.

We also performed ‘wet’ IFA for the anti-CD51/CD61 antibody. Blood samples from infected chicken were allowed to clot for 1 min, centrifuged at 3000g for 3 min to remove supernatant, and washed once with the same volume of 1X PBS. The washed blood cells were incubated with anti-CD51/CD61 antibody at RT for 2 h (1:200 dilution). The cells were washed with 1X PBS twice and incubated with secondary antibody solution containing Hoechst dye as described above. The cells were washed again, spread on a glass slide, and mounted for microscopic observation after air-dry at RT.

### Microscopy and confocal microscopy

The mounted IFA slides were observed under either a fluorescent microscope (Nikon Eclipse 50i) or laser-scanning confocal microscope (Zeiss LSM 780, Carl Zeiss).

The sealed slides were examined under 10X, 40X or 100X oil lens; images were captured and processed using ZEN2010 software or Photoshop software. Images were further processed using Adobe Photoshop and Adobe illustrator C6.

## Results

### Male and female gametocytes of *L*. *sabrazesi*

**Fig 1:**
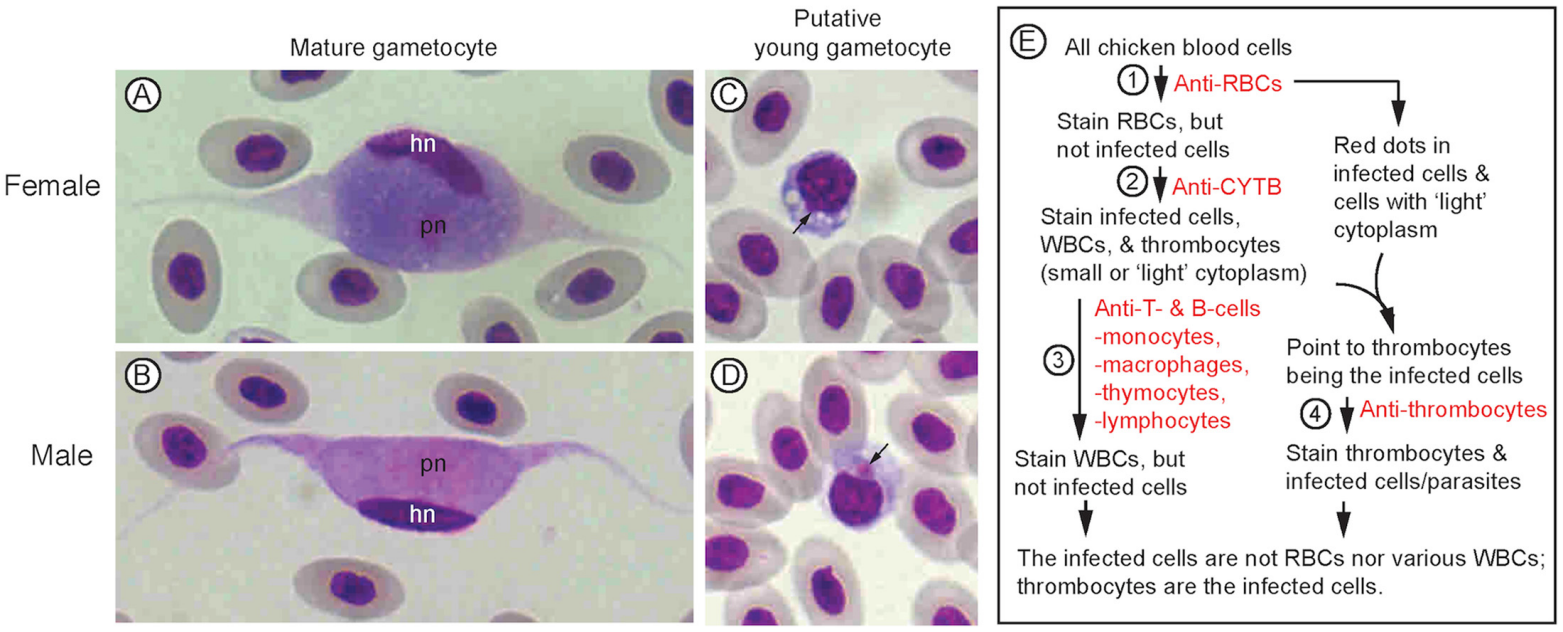
Gametocytes of *Leucocytozoon sabrazesi*. (A and B) Mature female and male gametocytes, showing elongated host cells (or parasites) with two long extensions. (C and D) Putative young female and male gametocytes within host cells having round nuclei that are slightly larger and stained darker than those of red blood cells. Arrows point to putative parasite nuclei. The blood smears were stained with Giemsa stain and observed under a microscope. hn, host cell nucleus; pn, parasite nucleus. (E) A flow chart of study design, antibodies used, and staining results from the antibodies. RBCs, red blood cells; WBCs, white blood cells.

Two species of *Leucocytozoon* (*L*. *sabrazesi* and *L*. *caulleryi*) were reported in Fujian Province of China based on the morphologies of gametocytes in blood smears [22, 23]. Mature *L*. *sabrazsi* gametocytes have fusiform morphology, whereas *L*. *caulleryi* have rounded gametocytes [1]. For samples collected from the Tzu Chi Chicken Farm, we only observed fusiform mature gametocytes (Fig 1A and 1B), suggesting that the parasites were *L*. *sabrazesi* gametocytes. Occasionally host cells with round nuclei may contain a red dot and blue cytoplasm with vacuoles likely representing a young female gametocyte (Fig 1C), or lightly stained cytoplasm with a red dot that may represent a young male gametocyte (Fig 1D). Additionally, our previous study comparing DNA sequences encoding mitochondrian *coxIII* and *cytb* suggested that the chickens in Haicang district, Xiamen, were infected with *L*. *sabrazesi* [21]. Therefore, the parasites in this study are considered to be *L*. *sabrazesi*. Because chicken RBCs are nucleated, it has been difficult to determine which host blood cell type(s), RBCs or any types of WBCs, the parasite resides. To identify the cell type(s) that *L*. *sabrazesi* gametocytes infect, we used various cell-type specific antibodies to stain host blood cells and the parasites. The major experiments performed in this study with brief results are summarized in Fig 1E.

### Anti-RBC antibodies do not stain the membrane of parasitized cells

**Fig 2:**
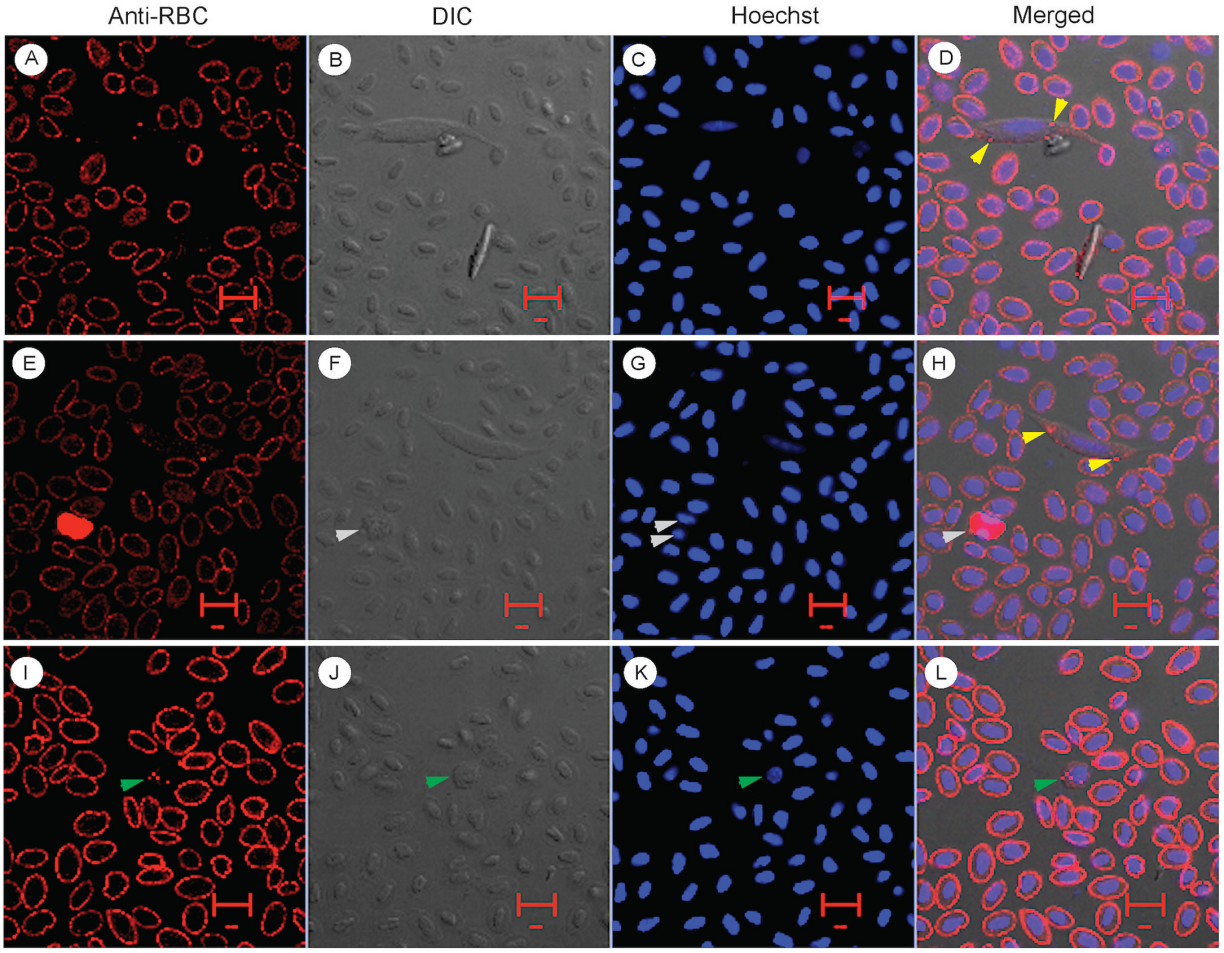
Confocal images of infected chicken blood cells stained with anti-red blood cell antibodies. Anti-RBC, images stained with rabbit anti-chicken red blood cell antibody 103–4139; DIC, differential interference contrast images; Hoechst, Hoechst dye staining of nuclei (DNA); Merged, merged images of anti-RBC, DIC, and DAPI. (A-H) Two cells infected with *L*. *sabrazesi* gametocytes have some red dots (yellow arrowheads) that appear to be within the cytoplasm of the host cells. (E-H) A strongly stained white cell (grey arrowheads) that has two nuclei and rough granules in the cytoplasm, suggesting heterophils, monocytes, macrophages, or eosinophils. (I-L) A small cell that has a round nucleus and red dots similar (green arrowheads) to those seen in the infected cells. The small size of the cell suggests that it is likely a thrombocyte. The red ruler in each image indicates 10 μm.

As discussed above, chicken blood cells are quite different from those of mammalian blood cells, including nucleated RBCs, granulated heterophils that are equivalent to mammalian neutrophils, and nucleated thrombocytes that functions as platelets. Avian blood also has monocyte, lymphocyte, eosinophil and basophil that are more similar to those of mammalian cells. Our first question was whether RBCs were the cells that the *L*. *sabrazesi* gametocytes infect. To answer this question, we stained infected chicken blood with rabbit anti-RBC polyclonal antibodies (103–4139, Rockland Immunochemicals, Gilbertsville, PA) and observed the stained RBCs under a confocal microscope. The antibodies stained RBC membrane, as anticipated, but not the membrane of the cells containing *L*. *sabrazesi* gametocytes (Fig 2A–2H). However, some small red dots were often found within the cytoplasm of the infected cell (yellow arrow heads in Fig 2D and 2H). The anti-RBC antibodies also strongly stained large granules within the cytoplasm of some WBCs that had segmented nuclei, suggesting monocytes, heterophils, or other leukocytes (Fig 2E–2H, grey arrowheads). Limited numbers of similar red dots could also be found in a type of cells that have round nuclei and non-granulated cytoplasm that are most likely thrombocytes (Fig 2I–2L; green arrowheads). The red dots are reminiscent of those seen in the infected cells (Fig 2D and 2H). The anti-RBC antibodies did not stain the membrane of the putative thrombocytes or the membranes of any other WBCs. The lack of membrane staining of the infected cells suggests that the parasite does not infect RBCs.

**Fig 3:**
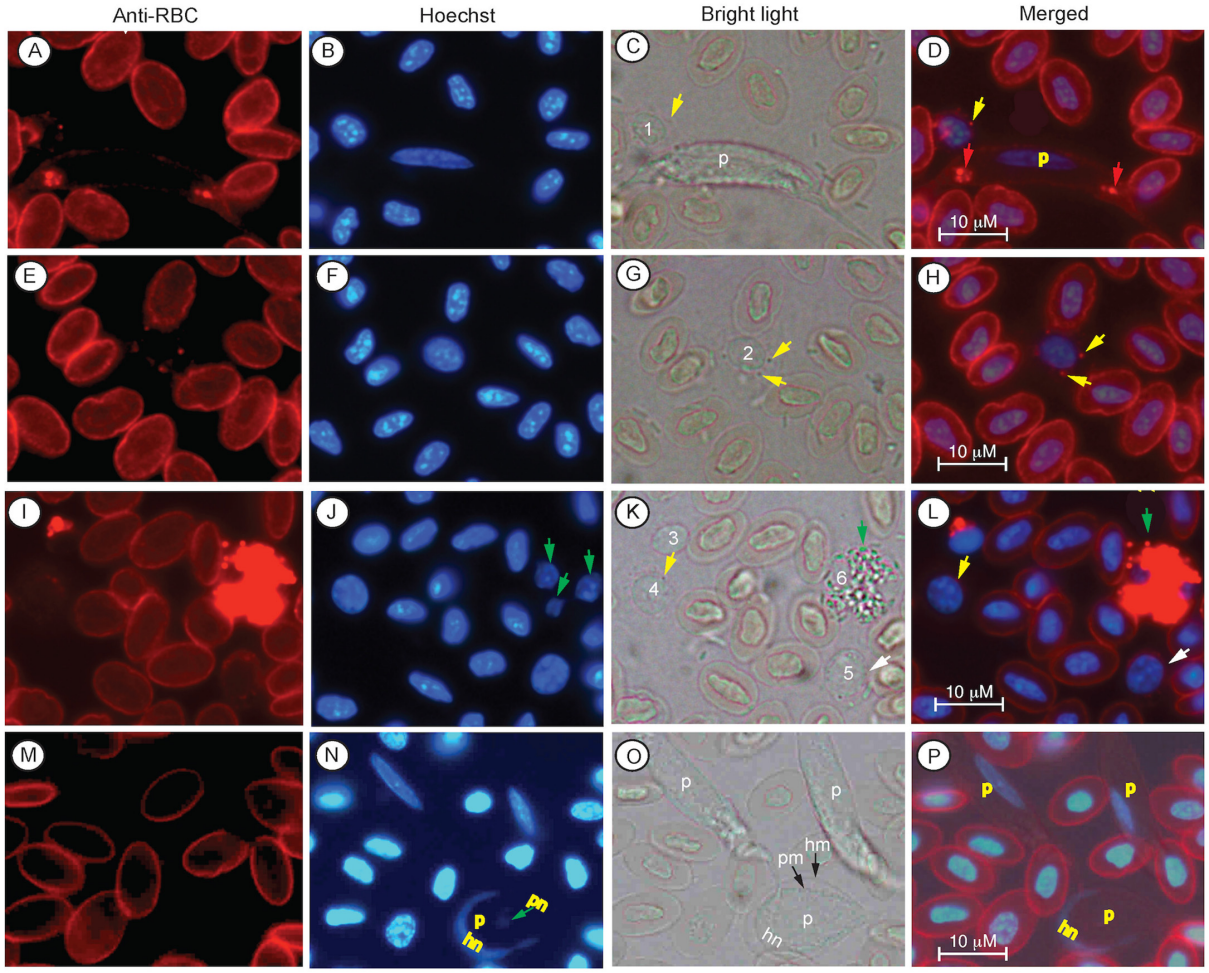
Images of infected chicken blood cells stained with anti-red blood cell antibodies and observed under a fluorescent microscope. Anti-RBC, images stained with anti-RBC antibodies; Hoechst, images stained by Hoechst dye; DIC, bright light images; Merged, merged images of the three. All staining and labeling are the same as those in Fig 2. (C, G, and K) Images under bright light show four cells (numbered 1–4) with relatively round nuclei and pale large cytoplasm with a few black dots under bright light or red dots after anti-RBC staining (yellow arrows). Number 5 indicates a cell that is likely a lymphocyte; number 6 is a cell possibly representing a heterophil; “p” indicates a gametocyte. (D) Red arrows point to red dots in a gametocyte-infected cell. Green arrows in J point to three nuclei of a white cell stained by anti-RBC (I and L). Images M-P show two mature gametocytes and one immature gametocyte (p = parasite). Host cell membrane (hm), parasite membrane (pm), and the host cell nucleus (hn) can be clearly seen (O and P), but not stained by the antibodies.

To better determine the cell types containing red dots after anti-RBC staining, we also observed the stained cells under a fluorescent microscope and bright light (Fig 3). The cells containing red dots had round or elliptical nuclei and generally had “clear” cytoplasm with limited numbers of fine dark granules or vacuoles (cells numbered 1–4 in Fig 3A–3L). These cells have the characteristics of immature thrombocytes, e.g. having a “clear” cytoplasm with some fine granules and/or vacuoles and a round or elliptical nucleus [9]. Again, the anti-RBC antibodies also stained the cytoplasm of some WBCs with large reflective granules, suggesting heterophils or eosinophils (Fig 3I–3L). The lack of staining by the anti-RBC antibodies of both infected host cell membrane and the parasites was clearly shown by an immature (oval) gametocyte having separated membranes of the host cell and parasite (Fig 3N–3P). We counted ~5,000 cells from 10 microscopic images (40X) from one infected chicken and found 10 gametocytes; none of the gametocyte-infected cells were stained by the anti-RBC antibody (Table 1). These results again suggest that *L*. *sabrazesi* gametocytes do not infect RBCs.

**Table 1:** Numbers of gametocytes and positive cells stained by various antibodies.

Antibody	No. images	No. cells	Gam. obs.	% Gam	No. pos. cell	% pos cells	Staining characteristics
Anti-RBC	10	5276	10	0.19	10	0.19	RBC, membrane; WBCs, granules; parasite, dots
Anti-CYTB	10	5973	8	0.13	130*	2.18	RBC, no; WBCs, granules; parasite, cytoplasmic
Anti-mono	10	3641	10	0.27	8	0.22	RBC, no; WBCs, granules; parasite, no
Anti-CD4	9	7854	12	0.15	6	0.08	RBC, no; WBCs, granules; parasite, no
Anti-Bu-1b	10	7712	10	0.13	8	0.10	RBC, no; WBCs, granules; parasite, no
Anti-CD51/61	34^#^	8498	2	0.02	99	1.17	RBC, no; thrombocytes; infected cells

*, among the 130 positive cells, 87 are putative mature thrombocytes, 40 are immature thrombocytes, and 3 cells have 2-lobe nuclei and granules.

All the cells strongly stained by the other antibodies also have 2-lobe nuclei and large granules in cytoplasm.

No. images, number of microscopic images examined; No. cells, numbers of cells counted; Gam. obs., numbers of gametocytes

observed. No. pos. cells, non-red blood cells stained by specific antibodies. “no” under staining characteristics indicates no staining.

^#^, Counted from ‘wet’ IFA (no fixation).

Many thrombocytes were in clusters even in anticoagulant solution; therefore, the counts were estimates. This chicken had very low parasitemia; two infected cells were found in the randomly selected fields.

Anti-mono = anti-monocyte.

### Anti-CYTB antibodies stain the parasite infected cells and thrombocytes

**Fig 4:**
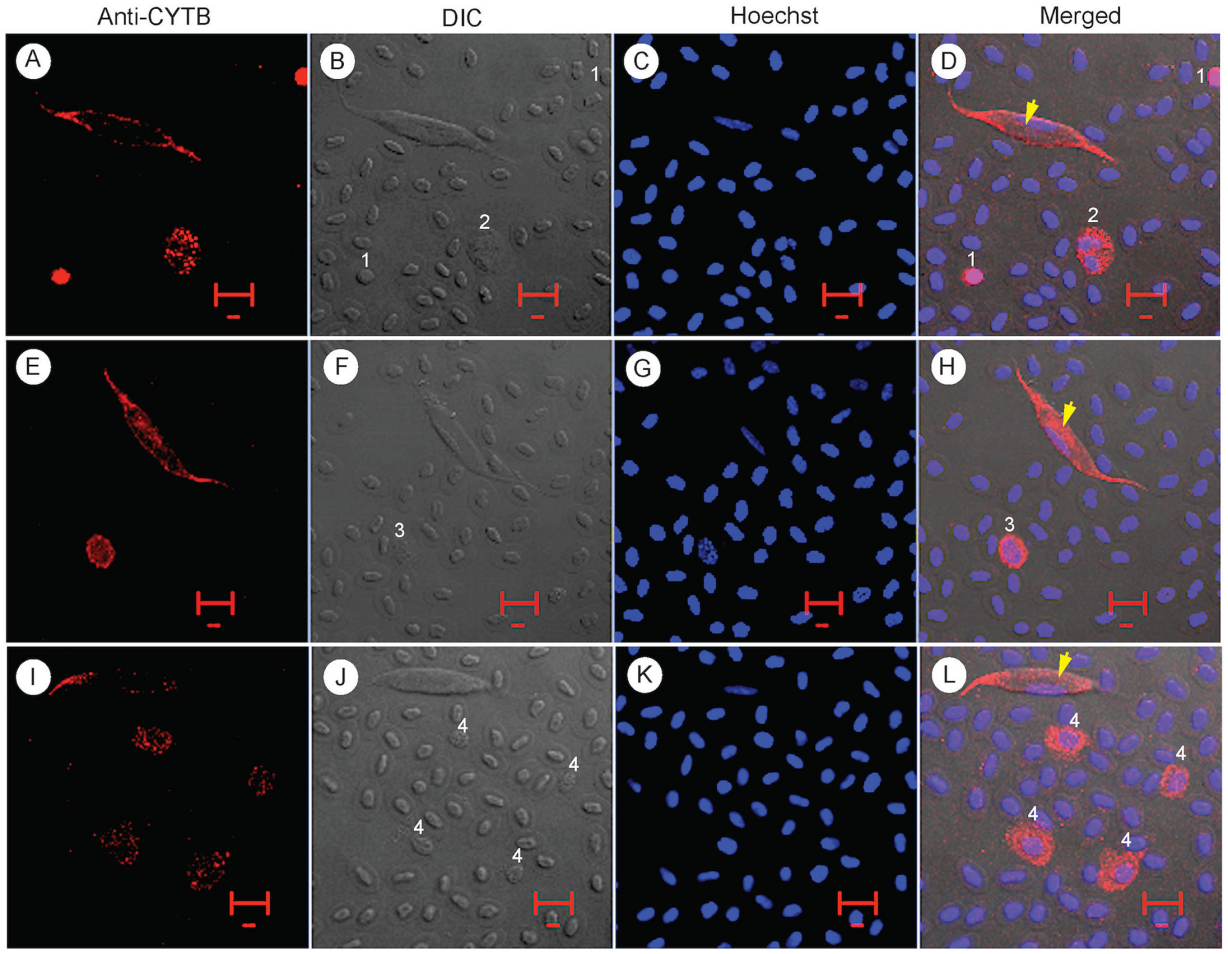
Confocal images of infected chicken blood cells after staining with anti-CYTB antibodies. Anti-CYTB, images stained with mouse anti-*L*. *sabrazesi* CYTB antibodies; DIC, differential interference contrast images; Hoechst, Hoechst dye staining cell nuclei (DNA); Merged, merged images of anti-RBC, DIC, and hoechst staining. The yellow arrowheads in (D, H, and L) point to three cells infected with *L*. *sabrazesi* gametocytes. Four types of recognizable host white cells are stained by the antibodies and are marked as 1–4: 1, likely mature thrombocyte; 2, monocyte/heterophil/neutrophil; 3, lymphocyte; 4, immature thrombocyte. The red ruler in each image indicates 10 μm.

**Fig 5:**
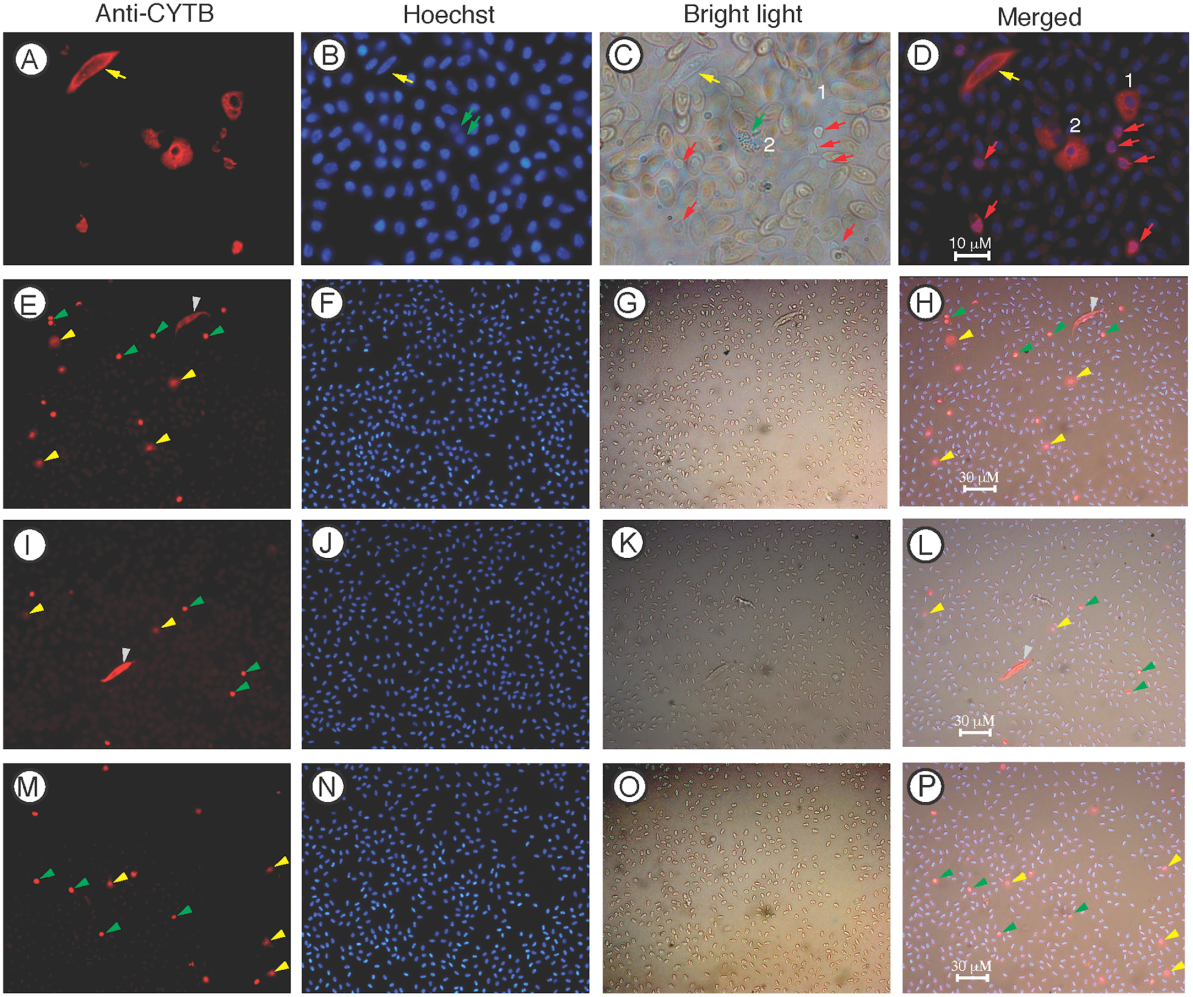
Images of infected chicken blood cells that are stained with anti-CYTB antibodies and observed under a regular fluorescent microscope. Anti-CYTB, Images stained with mouse anti-*L*. *sabrazesi* CYTB antibodies; Hoechst, image of Hoechst staining for nuclei (DNA); Bright light, bright light images; Merged, merged image of anti-CYTB, Hoechst, and bright light. Images (A-D) the yellow arrows point to a gametocyte; the green arrows indicate a white cell likely to be a heterophil with granules; the red arrows point to multiple putative mature thrombocytes; and number 1 and 2 mark two putative immature thrombocytes, with clear cytoplasm under bright light but stained by the antibodies. (E-P) images taken at 40X magnification. Yellow arrowheads point to putative immature thrombocytes; green arrowheads point to mature thrombocytes; grey arrowheads point to gametocytes. Note that a large number of putative thrombocytes were stained.

To generate antibodies against the parasites, we synthesized a peptide based on the predicted protein sequence of the *L*. *sabrazesi* cytochrome B (CYTB) we obtained in our previous study [21] and immunized mice to obtain polyclonal antibodies. Anti-sera from the immunized mice were found to stain infected cells and the parasites, as observed under a confocal microscope (Fig 4). The anti-CYTB antibodies also stained some uninfected WBCs, but not RBCs. Four types of WBCs could be stained by the anti-CYTB antisera, which could be partly due to non-specific antibodies generated against the carrier protein Keyhole limpet hemocyanin (KLH) because the peptide sequence is quite different from those of the host CYTB. The first type cell stained by the anti-CYTB was a small cell with round nucleus and with limited cytoplasm, suggesting mature thrombocytes (marked 1 in Fig 4B and 4D). The second type of cell (marked 2 in Fig 4B and 4D) was larger than RBCs and had granulated cytoplasm and two lobes of nuclei, suggesting heterophils or other leukocytes. The third type of cell appeared to be lymphocytes having a single large nucleus and limited cytoplasm (marked 3 in Fig 4F and 4H). The fourth cell type stained had round or oval nucleus and had fine granules strained by anti-CYTB antibodies (marked as 4 in Fig 4I and 4L). We also observed these cells under a regular fluorescence microscope (Fig 5A–5P). Under bright light, these cells had pale cytoplasm (marked 1 and 2 in Fig 5C and 5D) and also tend to appear together, suggesting immature thrombocytes. Although it appeared that multiple types of WBC were stained, anti-CYTB indeed predominantly stained mature and immature thrombocytes (Fig 5E–5P). From ~6,000 cells in 10 microscopic images taken at 40X magnification we counted 8 stained gametocytes, 87 strongly stained mature thrombocytes (round nuclei and limited cytoplasm), 40 stained immature thrombocytes (weaker stain and large cytoplasm), and only 3 putative heterophils (large cells with granules and two lobes of nuclei) (Table 1 and Fig 5E–5P). The staining patterns of anti-CYTB were consistent with the results from the anti-RBC staining, suggesting that the parasites infect a specific type of cells (likely thrombocytes), but not RBCs.

### The parasite does not infect heterophils, monocytes, macrophages or lymphocytes

Because the infected cells always had a single host nucleus, they are unlikely to be heterophils, eosinophils or even monocytes that generally have multi-lobe nuclei. To further investigate the specific type of WBCs the parasites infect, we performed additional IFA experiments using antibodies against different white cell lineages.

**Fig 6:**
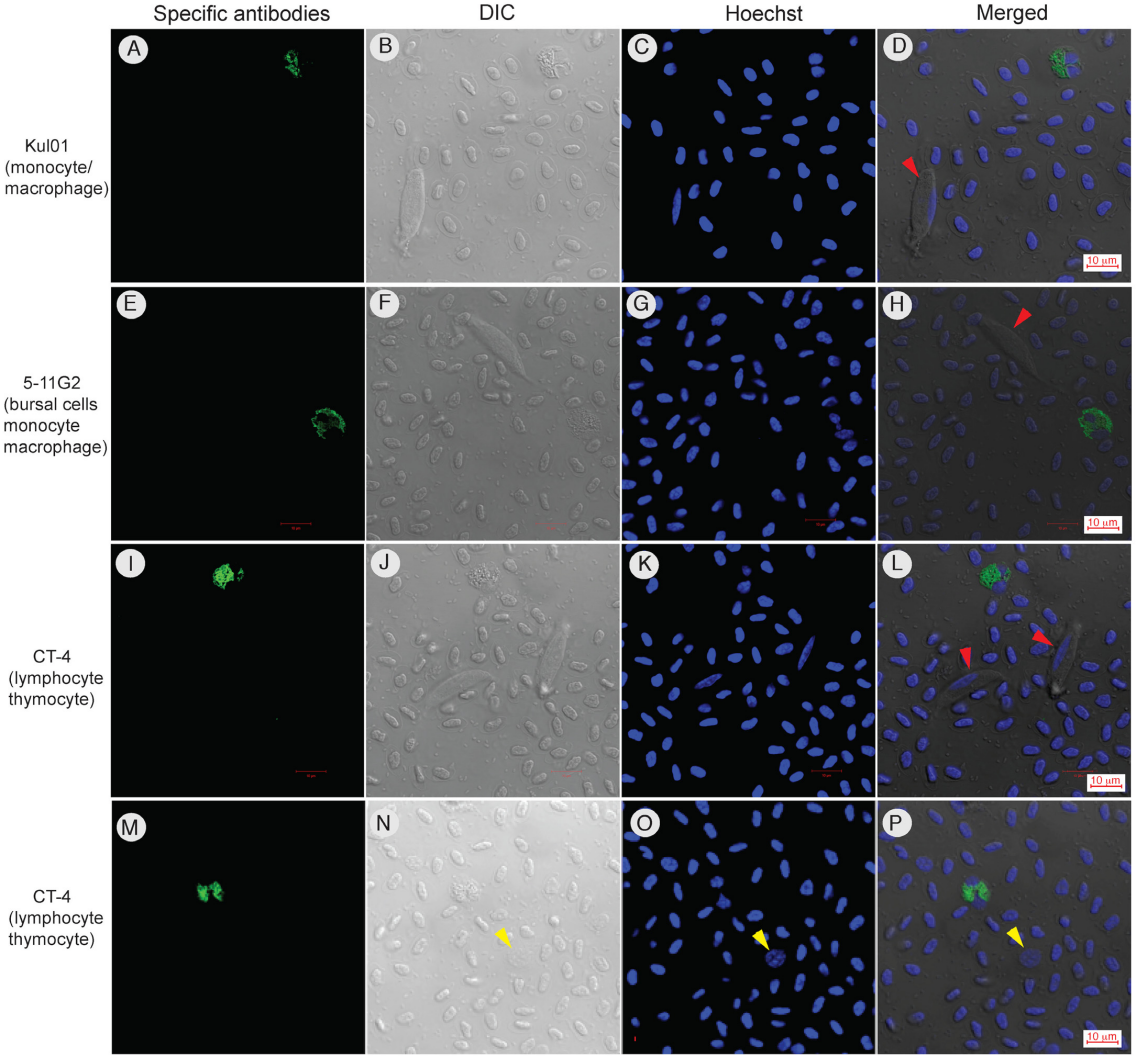
Confocal images of infected chiceken blood cells stained with cell lineage-specific antibodies. Green, images stained with specific antibodies (as labeled); DIC, differential interference contrast images; Hoechst, images of Hoechst dye staining for nuclei (DNA); Merged, merged images of anti-RBC, DIC, and Hoechst. (A-D) Cells stained with Anti-monocyte antibody that stains mostly monocytes and macrophages. (E-H) Cells stained with anti-Bu-1b (anti-chicken Bu-1b) that stains most bursal cells, thymocytes, and peripheral monocytes and macrophages. (I-P) Cells stained with anti-CD4 antibody (mouse anti-chicken CD4) that stains thymocytes, spleen cells, and peripheral lymphocytes. Cells infected with *L*. *sabrazesi* gametocytes (red arrowheads) are not stained by any of the three antibodies.

To rule out the possibility of monocyte being the cell type that *L*. *sabrazesi* infects, we used a monoclonal anti-monocyte antibody (KUL01, Southern Biotech, Birmingham, AL) to stain the chicken blood cells. The anti-monocyte antibody recognizes the mononuclear phagocyte system (MPS) and identifies chicken monocytes and macrophages as well as interdigitating cells and activated microglia cells, but does not react with B (Bu-1+) or T (CD3+) lymphocytes according to the product description from the antibody supplier. In our hands, the anti-monocyte antibody stained only a small number of cells that had one to two lobes of nuclei and had some large granules in their cytoplasm, but not the parasitized cells (Fig 6A–6D). We only counted eight cells stained by the anti-monocyte antibody and 10 unstained gametocytes from ~3,600 cells in 10 40X microscopic images (Table 1). These results rule out the possibility that monocytes or macrophages are cells infected by the parasite, consistent with the observation that infected cells always have one nucleus. Similar results were obtained when the chicken blood cells were stained with a monoclonal antibody anti-Bu-1b (5-11G2) that stains bursal cells, thymocytes, monocytes and macrophages, but not granulocytes, RBCs or thrombocytes. Again, the cells infected with the parasites were not stained by the anti-Bu-1b antibody (Fig 6E–6H). The cells stained by anti-Bu-1b were similar to those stained by anti-monocyte, mostly having two-lobe nuclei and some granules in the cytoplasm, and having similar frequency in the blood (8 stained cells and 10 unstained gametocytes from 10 images; Table 1). The results again suggest that bursal cells, thymocytes, monocytes and macrophages are not the cells the parasite infects.

We next stained the infected blood smears with anti-CD4 antibody that stains thymocytes (70%), spleen cells (10%), peripheral blood lymphocytes (45%), and bursal cells (<1%) (CT-4, Southern Biotech). In our hands, the anti-CD4 antibody stained cells were generally larger than RBCs, had segmented nuclei, and contained large rod-like granules. The appearance of the cells suggested that they were heterophils, eosinophils, monocytes, or macrophages (Fig 6I–6P). Again, the anti-CD4 antibody did not stain infected cells or the parasite. Based on cell size, granules in the cytoplasm, the number of lobes of nuclei, and the patterns of antibody staining, we can conclude that monocytes, macrophages, lymphocytes, heterophils, and eosinophils are not the infected cells. These results are also consistent with those from anti-CYTB and anti-RBC staining, suggesting that the cell type infected by the parasite is either thrombocytes or an unknown cell with single nucleus and pale cytoplasm.

### Anti-CD51/CD61 antibody specific for chicken thrombocytes also stains infected cells

**Fig 7:**
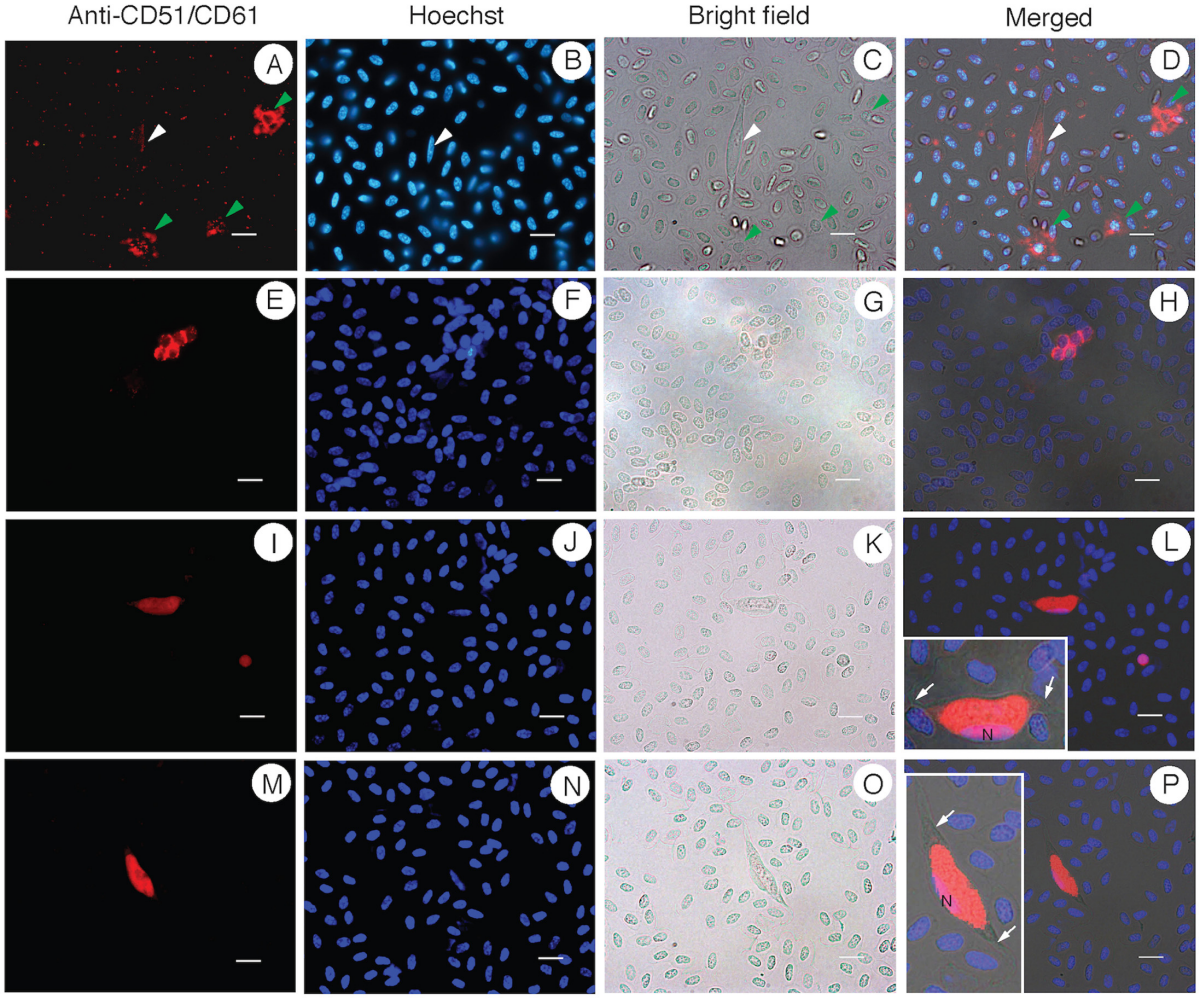
Images of infected chicken blood cells that are stained with anti-CD51/CD61 monoclonal antibody and observed under a regular fluorescent microscope. (A-D) stained cells with characteristic of thrombocytes having round nuclei and small pale cytoplasm (green arrows) and a weakly stained parasite-infected cell (white arrows). (E-H) A cluster of five thrombocytes stained red by the anti-CD51/CD61 antibody. (I-L) A stained parasite-infected cell and a stained thrombocyte (small red dot). (M-P) Another stained parasite-infected cell (red). Inset images of enlarged gametocytes in L and P show host cell membrane (arrows) and nuclei of host cells (marked ‘N’). Image A-D are from formaldehyde fixed cells; images E-P are from ‘wet’ IFA without fixation. The white scale bar is 10 μM.

The staining patterns of the above antibodies suggested that thrombocytes could be target cells that parasite infect. To confirm the identity of the infected cells, we searched the literature for antibodies specific for chicken thrombocytes and found a mouse monoclonal antibody against human CD51/CD61 (anti-CD51/CD61) that was shown to also specifically recognize chicken thrombocytes [24]. We performed IFAs using formaldehyde or cold methanol fixation methods and obtained weak (formaldehyde) or no staining (methanol) of the infected cells, although the antibody could stain thrombocytes (Fig 7A–7D). Considering that flow cytometry was used in the original report of the anti-CD51/CD61 antibody binding to chicken thrombocytes, we performed ‘wet’ IFA in liquid solution without fixation. We first tested the anti-CD51/CD61 antibody on cells from chicken blood allowed to clot for 1 min before washing the cells with PBS. Indeed, the ‘wet’ IFA experiments showed that the anti-CD51/CD61 antibody stained clustered cells with characteristic of thrombocytes (round nuclei with small pale cytoplasm) but not RBCs or other white cells (Fig 7E–7H). Importantly, the anti-CD51/CD61 antibody also stained parasite-infected cells (Fig 7I–7P). These results confirm that the cells infected by *L*. *sabrazesi* are thrombocytes, not RBCs or other white cells. Counting the anti-CD51/CD61-stained cells showed a frequency consistent with those stained by anti-CYTB antibodies. From 34 randomly selected microscopic fields, we counted 8,498 RBCs, 99 stained thrombocytes (~1.2%), and 2 infected cells (Table 1).

## Discussion

The question of which blood cell(s) *Leucocytozoon spp* gametocytes infect is more than a century old, and as we are aware of, there has been no specific study that can provide a definite answer to this question. Because the infected cells generally have a single nucleus, it has been difficult to determine the cell type(s) the parasite infects based on the morphology of the infected cells under a microscope. RBCs and/or mononuclear leukocytes have been considered the main targets that the *Leucocytozzon* gametocytes infect. The gametocytes of *Leucocytozoon spp*. were reported to develop in erythroblasts, RBCs, and mononuclear leukocytes (in cells of erythrocytic series and/or mononuclear leukocytes) [1]. Young gametocytes of *L*. *simondi* were described as found in lymphocytes, monocytes, myelocytes, and polychromatophilic erythroblasts, and macrophages [25]. However, cells infected by *L*. *fringillinarum* were believed to be in erythroblast, not WBCs [26]. In a more recent study using electron microscopy, Steele and Noblet considered *L*. *smithi* gametocytes being within monocytes or lymphocytes, but not RBCs or polymorphonuclear leukocytes [27]. The main reason for the difficulty in identifying the specific host cells infected by *Leucocytozoon* gametocytes is that the infected cells undergo rapid morphological changes after parasite invasion. Additionally, gametocytes of different *Leucocytozoon spp*. may infect different blood cells.

In this study, we used various antibodies to stain different types of chicken blood cells and showed that the cells infected by *L*. *sabrazesi* are not erythrocytes or leukocytes traditionally believed to be the host cells of *Leucocytozoon* gametocytes. Instead, we provide several lines of evidence to support the conclusion that *L*. *sabrazesi* gametocytes are in thrombocytes (likely invading young thrombocytes). First, the lack of staining of infected cells by anti-RBC antibodies rules out the possibility that gametocytes are within RBCs even though the antibodies stained the RBC membrane strongly. Second, the results from antibodies generated against parasite CYTB, despite not being specific for the parasite only, also support the conclusion that parasitized cells are not RBCs. The anti-CYTB antibodies did not stain RBCs, but predominantly stained cells with characteristics of immature and mature thrombocytes. The majority of the cells stained by the anti-CYTB antibodies had single round nuclei and had limited or “clear” cytoplasm (under bright light) that are characteristic of immature and mature thrombocytes. Third, the “red dots” within cytoplasm of putative thrombocytes and infected cells, when stained with anti-RBCs, also suggest common origin of the cells. Fourth, antibodies against monocytes, lymphocytes, macrophages, or bursal cells did not stain the infected cells, suggesting that these cells are not the infected host cells. And finally, a monoclonal antibody specific for chicken thrombocytes stained both thrombocytes and the infected cells. Based on these observations, we conclude that *L*. *Sabrazesi* gametocytes develop within thrombocytes, most likely young thrombocytes when the cells are in bone marrow.

The specific staining of thrombocytes and *L*. *sabrazesi-*infected cells by the anti-CD51/CD61 antibody provided the last piece of evidence confirming that *L*. *sabrazesi* gametocytes infect chicken thrombocytes. However, we should mention that the anti-CD51/CD61 antibody behaved differently under various IFA procedures. Fixation of the cells using cold methanol (-80°C) appeared to destroy the epitopes on the infected cells because no parasite-infected cells were stained under such condition (uninfected thrombocytes could be stained, data not shown). The results suggest that infection of parasite changes the structure or stability of the antibody targets on the infected cells. Similarly, formaldehyde could also affect the availability of the epitopes to the antibody, as the signals for the parasite-infected cells were greatly reduced after formaldehyde fixation. Only the procedure without any fixation produced strong and cell-type specific signals. The liquid (wet) IFA procedure is similar to the method used for flow cytometry analysis reported initially [24].

The frequency of thrombocytes in the blood is also consistent with the gametocytemia observed for the *Leucocytozoon* parasites. In our previous study, we found several chickens having *L*. *sabrazesi* gametocytotemia as high as ~0.3% (3 infected cells among 1,000 red blood cells) [21]. The frequency of ~1–2% cells stained by anti-CD51/CD61 (or anti-CYTB) is higher than the frequencies of cells stained by anti-CD4, Anti-monocyte or anti-Bu-1b antibodies (Table 1), although the number of WBCs can change after various infections or disorders. The relatively high frequency of thrombocytes in the blood can provide an adequate number of cells for the development of gametocytes. For some malaria parasites that infect RBCs such as *Plasmodium yoelii yoelii* YM, parasitemia higher than 50% are often observed [28]. If the *L*. *sabrazesi* gametocytotes infect RBCs, the gametocytotemia could potentially be higher than 1–2%.

Another unresolved issue is whether the “oval” gametocytes occasionally observed along with fusiform cells in some blood smears represent immature gametocytes or gametocytes of a different *Leucocytozoon* species. For *L*. *smithi*, quite conclusive evidence suggesting that the round gametocytes were young gametocytes was obtained after daily examination of tissue sections of laboratory-infected hosts [27]. The authors of the study also reported the presence of round gametocytes only in the deep circulation and the more elongated forms primarily in the peripheral circulation. This observation can explain the predominant elongated mature gametocytes in peripheral circulation in naturally infected birds. Interestingly, the immature gametocytes of *Plasmodium falciparum* malaria parasite were also reported to accumulate in the extravascular spaces of bone marrow [29, 30], suggesting that these Apicomplexan parasites share some common processes in gametocyte development. In this study, we also found predominantly fusiform mature gametocytes in blood smears from the infected chickens, except a few that appeared to be young gametocytes (Fig 1C and 1D; Fig 3N–3P). Based on the sequences of the genes encoding mitochondrial CYTB and COXIII obtained from the same Tzu Chi Chicken Farm that matched those of *L*. *sabrazesi* [21] and the predominant fusiform gametocytes in blood smears, we considered the parasites observed in this study being *L*. *sabrazesi*.

The identification of thrombocyte as the host cell infected by *L*. *sabrazesi* gametocytes raises some interestingly questions in the parasite’s sexual development. Have the merozoites already committed to sexual development when they infect thrombocytes or does the specific host cell provide signals that trigger the sexual differentiation? What are the specific ligands and receptors the sexually committed merozoites used during invasion if they are already committed to sexual development? Thrombocytes are relatively abundant in chicken blood [31], which provide sufficient opportunities for a parasite to infect and to ensure successful completion of its life cycle. In addition to promoting hemostasis in response to vascular injury by aggregating and releasing prothrombotic factors [14, 32], thrombocytes also play an important role in host immune response by secreting cytokines [16, 18, 33]. The invasion and destruction of thrombocytes by parasites may affect host blood clotting and/or the ability to respond to infections. Answering these questions will greatly enhance our understanding of the host-parasite interaction and the molecular basis of the disease. Additionally, a better understanding of the gametocyte development of *Leucocytozoon* parasites may provide important information for elucidating the molecular basis of sexual development of malaria parasites. Nonetheless, the identification of thrombocytes as the host cells that *L*. *sabrazesi* gametocytes infect answers a century-old question in *Leucocytozoon* biology and provides critical information for studying transmission and pathogenesis of the disease.
