# Correction: The Gametocytes of *Leucocytozoon sabrazesi* Infect Chicken Thrombocytes, Not Other Blood Cells

**DOI:** 10.1371/journal.pone.0137490

**Published:** 2015-08-28

**Authors:** The PLOS ONE Staff

There are errors in the Author Contributions. The publisher apologizes for the errors. The correct contributions are:

Conceived and designed the experiments: JLi X-zS. Performed the experiments: WZ JWL RX CZ QP XC LH YC. Analyzed the data: WZ JLi X-zS. Contributed reagents/materials/analysis tools: SL YC JY XL. Wrote the manuscript: JLi X-zS. Supervision of research: SL LH JY JLi X-zS.
